# Mathematics and the Brain: A Category Theoretical Approach to Go Beyond the Neural Correlates of Consciousness

**DOI:** 10.3390/e21121234

**Published:** 2019-12-17

**Authors:** Georg Northoff, Naotsugu Tsuchiya, Hayato Saigo

**Affiliations:** 1 Mental Health Centre, Zhejiang University School of Medicine, Hangzhou 310058, China; 2 Institute of Mental Health Research, University of Ottawa, Ottawa, ON K1Z 7K4 Canada; 3 Centre for Cognition and Brain Disorders, Hangzhou Normal University, Hangzhou 310036, China; 4 School of Psychological Sciences, Faculty of Medicine, Nursing and Health Sciences, Monash University, Melbourne, Victoria 3800, Australia; naotsugu.tsuchiya@monash.edu; 5 Turner Institute for Brain and Mental Health, Monash University, Melbourne, Victoria 3800, Australia; 6 Advanced Telecommunication Research, Soraku-gun, Kyoto 619-0288, Japan; 7 Center for Information and Neural Networks (CiNet), National Institute of Information and Communications Technology (NICT), Suita, Osaka 565-0871, Japan; 8 Nagahama Institute of Bio-Science and Technology, Nagahama 526-0829, Japan; harmoniahayato@gmail.com

**Keywords:** consciousness, mathematics, category theory, neural correlates of consciousness, integrated information theory, temporospatial theory of consciousness

## Abstract

Consciousness is a central issue in neuroscience, however, we still lack a formal framework that can address the nature of the relationship between consciousness and its physical substrates. In this review, we provide a novel mathematical framework of category theory (CT), in which we can define and study the sameness between different domains of phenomena such as consciousness and its neural substrates. CT was designed and developed to deal with the relationships between various domains of phenomena. We introduce three concepts of CT which include (i) category; (ii) inclusion functor and expansion functor; and, most importantly, (iii) natural transformation between the functors. Each of these mathematical concepts is related to specific features in the neural correlates of consciousness (NCC). In this novel framework, we will examine two of the major theories of consciousness, integrated information theory (IIT) of consciousness and temporospatial theory of consciousness (TTC). We conclude that CT, especially the application of the notion of natural transformation, highlights that we need to go beyond NCC and unravels questions that need to be addressed by any future neuroscientific theory of consciousness.

## 1. Introduction

“There is no certainty in sciences where mathematics cannot be applied”

(Leonardo da Vinci)

Consciousness has long been regarded as a mysterious phenomenon, and it has been mainly dealt with in philosophy. Past philosophers such as Descartes argued that consciousness is only accessible from the first-person perspective and cannot be explained from the third-person perspective. This tradition is followed by present philosophers who speak of an unbridgeable gap between the third-person physical objects of brain and first-person consciousness, formulated as the “explanatory gap problem” [1] or the “hard problem” [2] (see Part IV in [3] for a general overview). However, recent neuroscientific research postulates that consciousness is a biological phenomenon and the first-person perspective and phenomenal experience needs to be explained in a scientific framework [3,4,5,6,7].

The assumption of consciousness as a biological phenomenon has led neuroscience to search for the neural correlates of consciousness (NCC) [3,8,9,10,11,12]. The NCC has been defined as the minimum neuronal mechanisms jointly sufficient for any one specific conscious percept [11]. Recent progress in consciousness research introduces the following two refined interpretations of the NCC: (1) content-specific NCC, which determines a particular phenomenal distinction within an experience and (2) full NCC, which supports conscious experiences in their entirety, irrespective of the contents [13].

Major neuroscientific theories of consciousness, based on the empirical neuroscientific findings around the NCC, include the integrated information theory (IIT) [14,15], the global neuronal workspace theory (GNWT) [16,17,18,19] and most recently, the temporospatial theory of consciousness (TTC) [3,5,20,21]. Others include the higher order theories of consciousness [22,23], recurrent processing theory [24], operational space and time [25], neural synchrony [26], and social and attention schema theory [27]. Because the discussion of all these approaches is beyond the scope of this paper, we focus on two of the major theories, the integrated information theory (IIT) and temporospatial theory of consciousness (TTC).

The essential problem in our search for the NCC consists in bridging two domains of relationships, that is, relationships among the contents in conscious experience in the mental domain and relationships among neurons in the physical domain. One can thus speak of ”neurophenomenal relationship” connecting the brain’s neuronal states and the phenomenal features of consciousness [3,6]. One of the theories, IIT, for example, proposes the identity, which is one of the possible ontological relationships, “between experiences and conceptual structures” ([15], p. 11). Independent of how one frames the relationship in conceptual terms, theories about the NCC must address this fundamental problem about the relationships between physical and mental domains [28]. Transcending the empirical investigation of the neuronal states themselves, this requires mathematical tools to formalize the relationships between the two domains.

In consciousness research, there have been sporadic attempts to apply mathematical tools to bridge the gap between the physical and the mental domains [29,30,31,32,33,34,35]. However, tools such as graph theory, topology, algebra, and set theory are not sufficient to deal with the problem of consciousness. What is lacking in these mathematical tools is a strong mathematical formalization of relationships. Because the relationships are so fundamental in the physical and the mental domains, the mathematical tools that are built to deal with the relationships is the ideal tool for the studies of the NCC. In this review, we introduce a mathematical formalism, called category theory (CT). CT provides us with rich and mathematically well-developed classes of relationships, with natural transformation being the most important in this review.

Historically, CT was developed to establish and formalize relationships between different domains of knowledge that seem to differ in a fundamental way (for example, the mathematical fields of algebra and geometry) [36]. Such relationship could be established by introducing the notion of natural equivalence. Recently, CT has been proven extremely successful in connecting distinct domains of knowledge such as when unifying physics, topology, logic, and computation [37]. That renders CT a suitable mathematical candidate for consciousness research in its quest to formalize the relationship between two distinct domains, the physical and the phenomenal.

In fact, CT has been applied in neuroscience to memory [38,39,40] neural networks [41], perception [8], and cognition [42,43]. Going beyond a previous more general first attempt [28], in this review we propose that CT provides a useful mathematical framework for formalizing the neurophenomenal [3,6] relationship that underlies consciousness. For that purpose, we introduce three core concepts of CT including (i) category, (ii) inclusion functor and expansion functor, and (iii) natural transformation between them. Strategically, however, we will focus on dissecting the neuronal relationships, rather than address the neurophenomenal relationship directly.

One of the objectives of our paper is to provide a first step towards developing a mathematical formalization of the relationship between neuronal and phenomenal domains in the NCC. This will first be explicated on mathematical grounds and then applied to the NCC with IIT and TTC serving as paradigmatic test cases. Another objective is, through this exercise, to gain new insight into consciousness research, in particular, on the NCC, IIT, and TTC. We conclude that these CT-based concepts highlight similarities and complementarities in IIT and TTC. In particular, successful application of a natural transformation to IIT may open up a possible pathway to infer patterns of integrated information of a large system based on the patterns of integrated information of a subsystem that is a part of the larger system, which we tentatively term “reverse reductionism”. Furthermore, CT unravels and highlights several conceptual problems associated with content-specific NCC and full NCC, especially the consideration of essential roles played by natural transformation. In short, we point out that an exclusive focus on the relationship between one neuronal and one phenomenal state is unlikely to yield further fundamental progress in neuroscience of consciousness. Rather, we suggest that the focus should be on the relationships between different neuronal states and different phenomenal states. Such a shift of the focus will naturally lead to future neuroscientific theories of consciousness, which extend and go beyond the traditional concept of the NCC.

## 2. Category and Consciousness

### 2.1. Definition of Category

**Figure 1:**
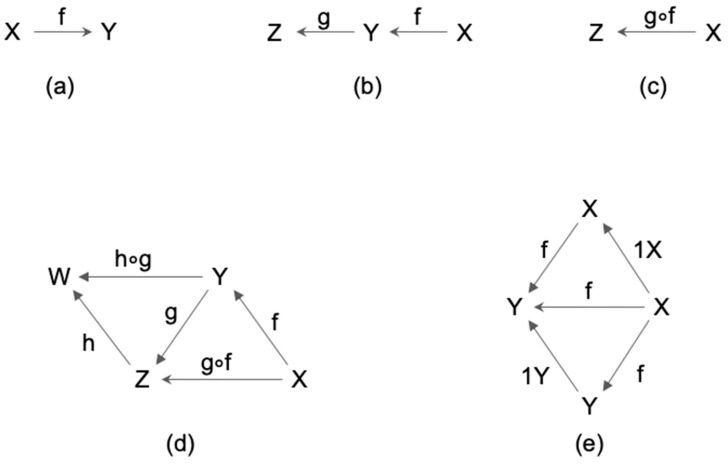
(**a**) Objects, arrows, domain, codomain: Each arrow f is associated with two objects, dom(f) and cod(f), which are called the domain and the codomain of (f). When dom(f) = X and cod(f) = Y, we denote f: X→Y, as shown in Figure 1a. (The direction of the arrow can be in any direction, from left to right or reverse, whichever is convenient.) A system with arrows and objects is called a diagram. (**b**) Composition: If there are two arrows f and g, such that cod(f) = dom(g), there is a unique arrow, (**c**) g ∘ f, called the composition of f and g. A diagram is called commutative when any compositions of arrows having the common codomain and domain are equal. (**d**) Associative law: (h ∘ g) ∘ f = h ∘ (g ∘ f). In other words, the diagram is commutative. (**e**) Unit law: For any object X there exists an arrow 1X: X→X, such that the diagram is commutative for any f: X→Y. In other words, f ∘ 1X = f = 1Y ∘ f for any f. 1X is called the identity of X.

A category is a system consisting of objects and arrows and satisfying the four conditions as shown in Figure 1.

By the natural correspondence from objects to their identities, we can identify an object (e.g., X) as its identity (e.g., 1X). In other words, we may consider objects are just special cases of arrows. This is one exemplar case where arrows play a more important role than objects in CT. In the following we sometimes adopt this viewpoint.

To sum up, the formal definition of a category is the following:

**Definition** **1.** A category is a system composed of two kinds of entities called objects and arrows, which are interrelated through the notion of domain and codomain, equipped with composition and identity, satisfying the associative and the unit law.

One of the strengths of the category theory is that it provides a unified formulation of sameness between different things, based on the notion of isomorphism, which is invertible arrows. More precisely:

**Definition** **2.** An arrow f: X→Y in a category C is called an isomorphism in C if there exists some arrow g: Y→X such that g ∘ f = 1X and f ∘ g = 1Y. Two objects are called isomorphic if there is some isomorphism between them.

Two isomorphic objects are essentially the same within the category. If X and Y are isomorphic and X are linked to some other objects through some arrow, then composition with the isomorphism provides the arrow from Y as well. Then Y can be considered as a version of X, which is the same as X, even when Y is completely different from X.

A famous isomorphism is the sameness between a donut and a coffee cup in topology. It actually means that they are isomorphic in a category Top, whose objects are topological spaces (a vast generalization of the notion of figures) and arrows are continuous maps, i.e., continuous transformations. We will use this notion of isomorphism in the following sections.

### 2.2. Category and Consciousness 

One of the most fundamental problems in consciousness research is to clarify the relationship between the neuronal and the phenomenal domains, the neurophenomenal relationship as stated by TTC [3,21].

From the CT viewpoint, the phenomenal domain can be formulated as a category whose objects are contents of consciousness as experienced and arrows are relationships between them as experienced. Let us call this category the phenomenal category and denote it as P.

The formulation of the neuronal category turns out to be problematic, which is the reason why we focus on the NCC in this paper. To see the nature of the problem, consider the representative neuroscientific approach to the problem of consciousness, to identify the neural correlates of consciousness (NCC). Content-specific NCC are usually defined as “the minimum neuronal mechanisms jointly sufficient for any one specific conscious percept” [13]. This definition is vague as to whether ”neuronal mechanisms” mean either the anatomical structure or the activity states of the neurons in the relevant mechanisms. Typically, the anatomical structure is assumed to be one that is usually found in the healthy brains of adult humans who can introspectively report their contents of consciousness with accuracy. Under such an anatomical assumption, a pattern of neural activity in a specific anatomical location over a certain temporal period is usually considered to be content-specific NCC. An example of a face-related NCC is the extended neural activation infusiform gyrus in the right hemisphere [44,45,46,47]. If the activity in this area is transiently lost due to electrical stimulation, face perception gets disrupted without affecting other types of percept [48].

A traditional NCC approach can be described as a research paradigm, where a snapshot of the pattern of neural activity, N, is minimally sufficient for a specific conscious phenomenology P. For example, Chalmers (2000) wrote, as one potential way to define the NCC for an arbitrary phenomenal property P, as follows:

“A neural correlate of a phenomenal family S is a neural system N such that the state of N directly correlates with the subject’s phenomenal property in S.”

From the CT perspective, this approach can be rephrased as the following: First, it tries to identify the neuronal category N as the category whose objects are patterns of neuronal activities in a specific region of the brain and whose arrows are transitional relationships between the patterns of neuronal activities. The phenomenal category P can be considered with its objects content of consciousness and with arrows transitional relationships. Second, it tries to find a sufficiently strong correlational relationship between the regions’ neuronal activities, for example, its neuronal state, and the category of consciousness, regarding it as the NCC.

While this approach seems quite natural, it has several difficulties, as pointed out by others (e.g., see [49]). One of the fundamental issues is that the neural activity pattern, N, needs to be defined within some anatomical reference frame. For example, face perception is typically correlated with the neural activity in fusiform face area (FFA) in normal healthy subjects. However, brain-damaged patients, whose damage spares a more or less normal level of neural activity in FFA, can be impaired in face perception [50]. Considering even more extreme cases, almost nobody would argue that a conscious face phenomenology, p, arises from the neural activity pattern N within FFA, which are artificially cut from the rest of the brain and kept alive and functional in a jar. Even if such an entity were to experience consciousness, unlike normal healthy humans, it would not experience it as a visual face phenomenology because it does not have any capabilities to experience other possible phenomenologies to compare with [51,52].

To summarize, most traditional NCC approaches implicitly require that the NCC is embedded in some anatomical reference frame that extends beyond a single region as the NCC. This entails that the neuronal activities of two, if not more, regions will serve as the NCC, which renders problematic the assumption of a single neuronal state, N, serving as the NCC. We must consequently raise the question of the exact relationship between the anatomical reference frame, for example, different regions and the neural activity patterns, for example, the neuronal states. In this paper, to address this issue, we propose to consider the “relationship” between at least two neuronal categories, N0 and N1, instead of one single category N. In short, we consider N0 as the actual state of the neural activity of the actual network, and N1 as all possible states of the neural activity of all possible networks. (To clarify, when we say that N0 is actual at a given moment, we are considering the actual anatomical structure, which includes strength of stochastic connections between elements (e.g., synaptic connections among neurons), which determines the transition probability matrix and the momentary dynamics of the system. Furthermore, N0 also specifies the actual activity pattern that the network is in at that given moment. While N1 includes all possible anatomical structures with all possible activity patterns, the ones that directly determine the NCC are those that ae related to N0 (actual). Note also that we do not refer to NCC as either actual or possible. Rather, we argue that the NCC should be considered as the relationship between N0 AND N1. The relationship between N0 AND N1 jointly determines its relationship to the phenomenal domain, P.) As we argue, considering how the actual network state and structure (N0) is embedded in a larger context of all possible network states and structures (N1) [52], in turn, that will allow us to clarify why we need to consider the anatomical reference frame to consider the NCC. This allows us to reconsider the relationship between the neural and the phenomenal categories in a more nuanced way and consequently to account for the phenomenal features of consciousness in a more comprehensive way. In the next section we will give a more detailed explanation of the way to conceive how the two categories, N0 and N1, are related to the phenomenal category, P, in the context of IIT and TTC.

### 2.3. Categories in IIT and TTC

IIT and TTC theories conceive a more complex notion of content-specific NCC that extends and goes beyond the assumption of a single neuronal state, for example, category N, serving as content-specific NCC. Thus, these theories agree that we need to introduce at least two neuronal categories (e.g., N0 and N1) to explain content-specific NCC, however, IIT and TTC differ in the exact formulation of the two neuronal categories. Note, here, we do not go into details of IIT and TTC and instead we focus on those aspects that are relevant within the present category theoretical approach.

#### 2.3.1. Categories in IIT

For the full description of IIT, see [14,15,53] (We note that CT analysis of IIT, in and of itself alone, is highly unlikely to help solve many known difficulties in the calculation of integrated information in IIT (e.g., finding the minimum partition of a system which is composed of many elements). For this type of specific problem, specific mathematical analysis and invention is necessary (e.g., [54,55]). Instead, what CT offers are more abstract, yet potentially more widespread and high-impact, problem solutions, as we elaborate later on (e.g., the reverse reductionistic approach)). Briefly, IIT starts from identifying the essential properties of phenomenology (existence, composition, information, integration, and exclusion [53]) and then claim phenomenology is “identical” to the conceptual structures. Then IIT proposes several postulates based on which types of physical mechanisms could potentially support such conceptual structures.

One essential aspect of IIT is that rather than focusing on only the actual state of a set of neurons, it considers the relationship between all possible states and an actual state of the set. Any conscious experience is informative in the sense that it specifies one of many possible experiences. Furthermore, IIT considers how a system (or a mechanism) is potentially affected when the system is disconnected in all possible ways. In other words, IIT considers the relationship between all possible network configurations and an actual network configuration.

The original IIT can be regarded to propose a relationship between conscious experience (or phenomenal category, P), conceptual structure (or informational structure category, I), and physical substrates (or neural category, N), where P is “identical” to I [14,53]. One way to view IIT is a functor from N to I (see Functors and natural transformations in IIT and TTC, Section 3.3). So far, IIT just assumes that I is “identical” to P. (IIT starts with the assumption that I, which is called maximally irreducible conceptual structure (MICS) in IIT, and P are identical [53]. In CT, the term “identity” has a very strict and well-defined meaning, and an “identity” relationship in CT sense is highly unlikely to be applicable between MICS and P. Mathematically, the assumption of the identity is way too strong. We believe the existence of functor from MICS to P, and from P to MICS as well, are reasonable to expect (and can be empirically tested through experiment [56]. In addition, adjunction is likely to exist between them (for those other concepts in CT, see [57]). With another advanced concept of categorical equivalence (which we will not go into the details in this paper), P may be shown to be categorically equivalent with MICS. As a possibly most strong relationship, we can expect P and MICS to be categorically isomorphic [58], where starting from MICS to go to P, we can always come back to the same MICS (and also starting from P to MICS and then back to P), but above and beyond this (e.g., identity) is not possible to test scientifically and mathematically. Isomorphic categories are usually “not” identical.) Future work is needed to investigate the detailed formulation and analysis on the structure of I or P (Tsuchiya and Saigo, in preparation). Thus, in this paper, we will focus on how IIT treats the category of N through an IIT functor and a possible IIT natural transformation (as we introduce in functor, natural transformation, and consciousness) and demonstrate that rigorous, yet complex, operations of IIT [53] can be reinterpreted through CT, which eventually offers a fresh and interesting insight on a potential reverse reductionistic approach in IIT.

**Figure 2:**
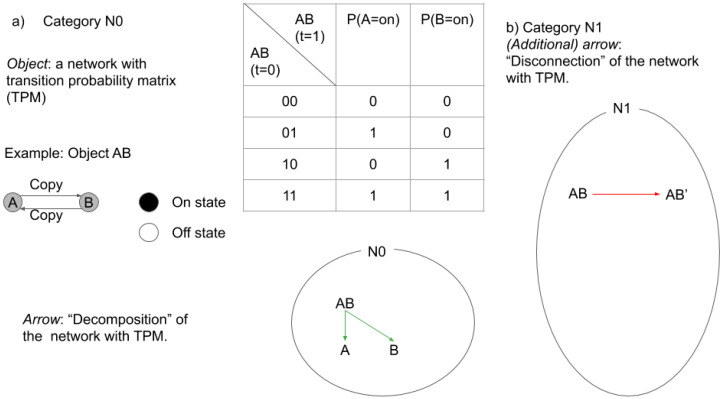
(**a**) In integrated information theory (IIT), category is defined by an object that is a stochastic network with transition probability matrix (TPM). The exemplar network is composed of a copy gate A and B, which copies the state of the other gate with a time delay of 1. The state of the gate is either on or off. The table on the right describes its TPM. An arrow in category N0 is “decomposition” of the network with TPM (Note that we grossed over various details that are important for IIT3.0 (e.g., distinction between past and future). In particular, how decomposed subnetwork should be embedded with the original network requires careful consideration of so-called “purview” in IIT3.0. Within the IIT’s algorithm, what we call “decomposition” corresponds to a step where one evaluates all potential candidate *φ* or small phi. For example, for a system ABC, its power set, A, B, C, AB, BC, AC, ABC needs to be evaluated. In some cases, decomposed candidate small phis may not exist, thus it may better be called as “potential decomposition”. However, for simplicity, we prefer to call it as “decomposition”.). Decomposition allows IIT to quantify the causal contribution of a part of the system to the whole. (**b**) Disconnection arrows find the minimally disconnected network, which captures the concept of the amount of integration in IIT.

Let us start considering what are the essential categories in IIT and what corresponds to objects and arrows in category N0 and N1 in IIT. (Note that we grossed over various details that are important for IIT3.0 (e.g., distinction between past and future). In particular, how decomposed subnetwork should be embedded with the original network requires careful consideration of so-called “purview” in IIT3.0. Within the IIT’s algorithm, what we call “decomposition” corresponds to a step where one evaluates all potential candidate *φ* or small phi. For example, for a system ABC, its power set, A, B, C, AB, BC, AC, and ABC needs to be evaluated. In some cases, decomposed candidate small phis may not exist, and thus it may better be called “potential decomposition”. However, for simplicity, we prefer to call it “decomposition”.) We propose that in IIT category an object is a stochastic causal network with transition probability matrix (TPM) to describe its state transition and an arrow is a manipulation on the network with the TPM (Figure 2). IIT considers various rules for the types of manipulations and selections of arrows, however, these manipulations can be relaxed or compared with various other types which could be an informative research direction in its own. For our purpose, it is important to note that IIT considers configurations of causal relationships by quantifying how each powerset of mechanism contributes to the whole. IIT does this by introducing an arrow that we call decomposition. Decomposition operation can be considered as something similar to marginalization. The purpose of the decomposition operation is to consider and quantify how much a neuron, A, contributes to a system of neurons, A and B. Thus, given an object AB, we have at least three arrows as follows: AB→AB (identity), AB→A, and AB→B. Decomposition arrows capture one of the central properties of IIT, that is, axiom and postulate of “composition” in consciousness.

Note, N0 satisfies all the requirement to be a category (identity, associativity, and compositionality are all satisfied).

Next, consider a category, N1, in which objects are all possible networks associated with TPM. N1’s arrows are decomposition as in N0 and also disconnection. Disconnection operation can be considered as transformation of TPM to another TPM through (virtual) disconnection of the network, such that subsets of the network are statistically independent [59,60]. Disconnection arrows capture another central property of IIT, that is, axiom and postulate of “integration” in consciousness. The disconnection arrow can be related to the amount of integrated information.

Again, note that N1 also satisfies all the requirements to be a category. In functor, natural transformation, and consciousness, we will discuss how these categories are related through functors.

#### 2.3.2. Categories in TTC

Unlike IIT, the TTC does not consider different neurons’ or regions’ activities as starting point to distinguish different neuronal states. Instead, TTC stresses the temporal dimension, and thus the dynamics of neuronal activity as it operates across different regions and points in time (for details, see [3,5,6,12,20,21,61,62]). Specifically, for the TTC, N0 and N1 are the temporal dynamics of neural systems (extending possibly across all brain areas). N0 precedes N1 in time. In other words, N0 can be regarded as prestimulus (which ultimately can be traced to the continuously ongoing dynamics of the spontaneous activity) and N1 as poststimulus neural activity.

Note, we operationally distinguish pre- (N0) and poststimulus (N1) activity in order to empirically consider a case where some stimulus is consciously perceived by someone. Here, prestimulus activity refers to the ongoing dynamics prior to its modulation by any specific stimulus. In contrast, poststimulus activity describes the activity following the onset of a specific stimulus, this activity contains the activity evoked by the stimulus itself and the ongoing dynamics, with the latter overlapping from the pre- into the poststimulus interval. Importantly, both components, internal prepoststimulus ongoing dynamics and poststimulus evoked activity related to the external stimulus interact in a dynamical, for example, non-additive, way (see below, [63,64,65]). To support the claim of the dynamical interaction of internal pre and post ongoing dynamics and external stimulus, we consider the empirical data in fMRI [66,67,68,69] and EEG and MEG [70,71,72,73]. These data show that the amplitude and variance of prestimulus activity plays a major role in whether the subsequent stimulus and its respective contents becomes conscious or not. Typically, high prestimulus activity levels, e.g., high amplitude or variance, are more likely to allow for associating contents with consciousness than low prestimulus activity levels. Baria and colleagues [72] showed that the prestimulus activity level, up to 1.8 s prior to stimulus onset, can predict (on a single trial level, above chance) whether a visual content will be consciously seen or not. Moreover, prestimulus activity levels are not only relevant in the region typically processing the respective stimulus, for example, similar to FFA for face stimuli and auditory cortex for auditory stimuli, etc. Additionally, the prestimulus activity level in other more distant regions like parietal and prefrontal cortex have also been shown to be relevant for impacting conscious perception of an object during the poststimulus period [66,67,68,69].

Together, these data suggest that both prestimulus activity levels, for example, amplitude and variance, and poststimulus activity level may need to be included in content-specific NCC. Specifically, as emphasized by TTC, it is the temporal and spatial dynamics of the prestimulus activity, for example, its variance being present in different regions, that is central for associating poststimulus activity and its contents with consciousness (see below for more details on the pre- and poststimulus dynamics and how it allows for a particular visual stimulus to be consciously perceived).

Accordingly, the TTC entails a more complex notion in content-specific NCC, which extends and goes beyond a single neuronal state (and thus also beyond the neural prerequisites of consciousness, [8,10]) when assuming the temporospatial dynamics of two distinct neuronal states to underlie consciousness. Mathematically, that requires two distinct neuronal categories, i.e., N0 and N1 in order to formalize content-specific NCC of TTC within the context of CT.

To be more explicit, for TTC, objects of N0 and N1 are neural activity over time and space, and arrows are explicitly defined only for identity. This guarantees that N0 and N1 are both categories.

As a critical component to consider consciousness, TTC considers temporal dynamics of pre- (N0) and poststimulus (N1) neural activities as objects of these categories. Therefore, TTC is compatible with a dynamic systems approach that emphasizes attractor and “dynamical activity space trajectories” as distinguished from single points in time and space (as we perceive and cognize them) [35,74]. Such dynamical structure, for example, “dynamical trajectory space” [34], is assumed to account for consciousness and, more specifically, the phenomenal features of consciousness [21,30,74]. Framed in the context of CT and its focus on natural transformation, TTC claims that temporospatial dynamic is central for transforming neural activity, e.g., N0 and N1, into phenomenal features, for example, P.

In summary, although the specifics are different, IIT and TTC are clearly going beyond a traditional NCC conceptualization, a particular neural state at a given time N to correspond to a particular phenomenal state, P. Rather, IIT and TTC both point to it as a relationship between N0 and N1 that corresponds to a particular phenomenal state, P. In the next section, we will introduce a mathematical tool to consider a relationship between the two categories, that is, functor.

## 3. Functor, Natural Transformation, and Consciousness

### 3.1. Definition of Functor and Natural Transformation

**Figure 3:**
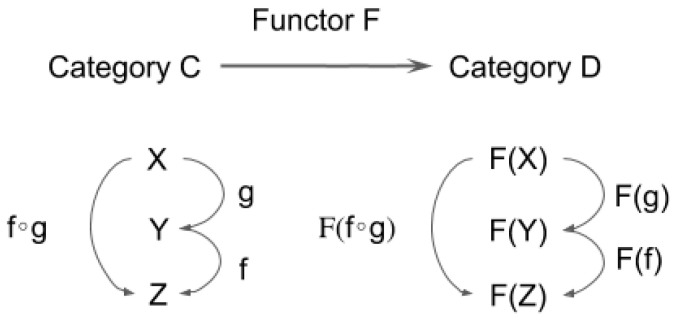
Schematic depiction of a functor: a structure-preserving mapping from one category to another category.

A functor is defined as a structure-preserving transformation between two categories. In fact, a functor is defined as an arrow in “the category of categories”, shown in Figure 3 below.

**Definition** **3.** *A correspondence F from a category C to another category D which maps each object and arrow in C to a corresponding object and arrow in D is called a functor if it satisfies the following 3 conditions:*

*1.*  It maps f: X→Y in C to F(f): F(X)→F(Y) in D;
*2.*  F(f ∘ g) = F(f) ∘ F(g) for any (composable) pair of f and g in C;
*3.*  For each X in C, F(1X) = 1F(X).

In short, a functor is a correspondence which preserves categorical structure. Through a functor, one category and its associated structure is related to those in another category and its associated structure. A functor allows us to consider the possibility to relate obviously different domains (e.g., the phenomenal and the neuronal) to each other.

**Figure 4:**
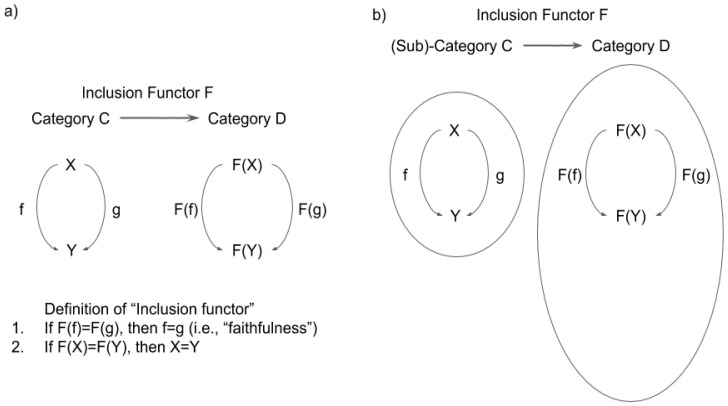
(**a**) Definition of “inclusion functor”. (**b**) Subcategory C is included by category D if inclusion functor F: C->D exists. Note that C does not need to be “a part of” D to be “included” (unlike a commonsense definition of “inclusion”).

One of the most important notions that we introduce in the present paper is what we describe as an “inclusion functor” (Figure 4a). Let us consider two categories *C* and *D.* A functor F from C to D is called an inclusion functor if:

For any pair of object X, Y in C and arrows f, g in C from X to Y, i.e., dom(f) = dom(g) = X and cod(f) = cod(g) = Y, F(f) = F(g) implies f = g. (Functors satisfying this condition are called “faithful’’.)

For any pair of objects X and Y in C, F(X) = F(Y) implies X = Y.

When there is an inclusion functor from *C* to *D*, *C* is called a subcategory of *D*. (From a more radically category theoretical viewpoint, the inclusion functor F itself is called subcategory.)

The intuition for the terms can be explained as follows (also see Figure 3): Let us consider the situation that any object and arrow in *C* has a corresponding object and arrow in *D*, and the notion of dom and cod, composition, and identity for *C* are in common with those for *D*. Then it is quite natural to think of *C* as a subsystem of *D,* and thus to call *C* a subcategory of *D.* In this situation, we can define an inclusion functor F as a map sending each object and arrow in *C* as an object and arrow in *D*, i.e., F(X) = X and F(f) = f for any object X and arrow f (Here X and f in the left hand side are an object and an arrow in *C* and those in the right hand side are those considered in *D*).

**Figure 5:**
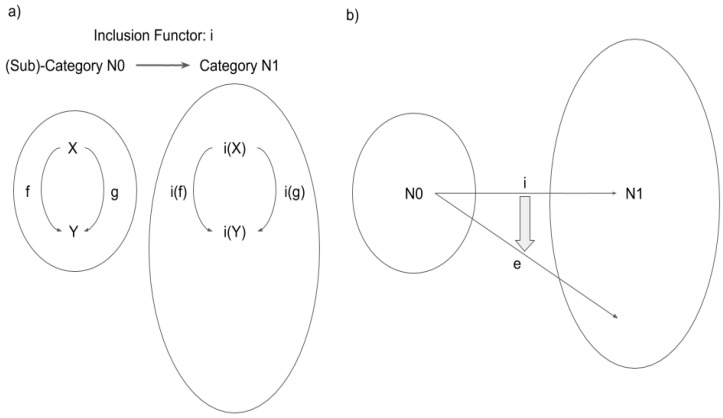
(**a**) Inclusion Functor i: N0→N1. N0 is included in N1 through Inclusion Functor i. (**b**) Expansion Functor e: N0→N1. e is a different structure preserving mapping from N0 to N1 (i.e., a functor from N0 to N1), but there is “natural transformation” from i to e.

Let us briefly summarize the meaning of the inclusion functor. The existence of the inclusion functor from N0 to N1 essentially means that N0 is a “subcategory” of N1. An inclusion functor i plays a fundamental role in this paper. i is defined by i(X) = X and i(f) = f (Figure 5). It works as the “basis” of the consciousness phenomena.

To define the notion of expansion functor, which is a functor different from inclusion functor but closely related to it, we need to define the notion of “natural transformation” as a relation between functors.

A functor is an “arrow” between two categories, but a functor can also be considered an object in CT (as an arrow can be considered an object, see definition of category, Section 2.1). When we consider functors themselves as “objects”, we call “arrows” between functors “natural transformations”.

**Figure 6:**
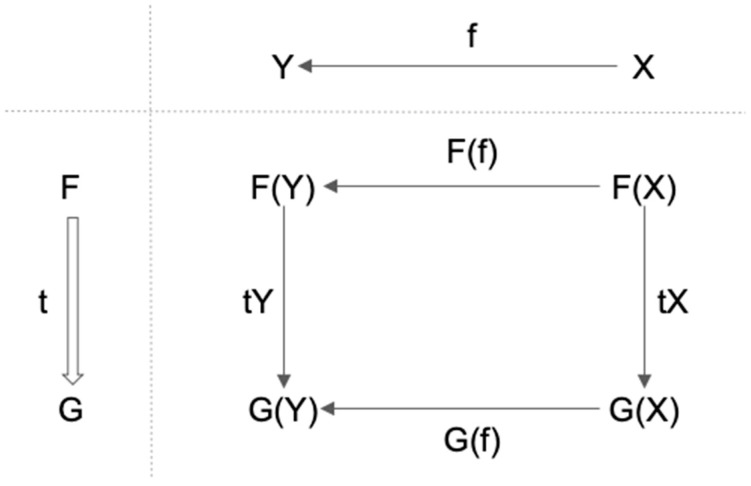
Schematic depiction of a natural transformation: a structure-preserving mapping from one functor to another functor.

The definition of natural transformations is the following (Figure 6):

**Definition** **4.** *Let F, G be functors from category C to category D, a correspondence t is called a natural transformation from F to G if it satisfies the following two conditions:*

*1.*  t maps each object X in C to corresponding arrow tX: F(X)→G(X) in D;
*2.*  For any f: X→Y in C, tY ∘ F(f) = G(f) ∘ tX.

For the natural transformation, we use the notation such as t: F⇒G. In Figure 6, the upper-right part denotes the arrow in C (f: *X→Y*). The lower-left part denotes the natural transformation from F to G (t: F⇒G). The second condition in the definition of natural transformation means that the diagram in the lower-right part commutes.

Intuitively speaking, a natural transformation from functor F to functor G is the system of arrows indexed by objects, which satisfies certain consistency conditions. This is an interesting property of CT and it is one of the most important concepts we introduce to consciousness research in this paper. A meta-level and abstract concept of a natural transformation is represented as a set of lower-level and concrete concept of arrows in a category. (We believe this nested mathematical structure of CT is particularly suited to capture some structural properties of the domain of phenomenology, P, which we will describe elsewhere.)

Now, equipped with this notion of a natural transformation, we can talk about a structure preserving map between two functors. Now, we introduce the notion of an expansion functor, as a functor towards which there is a natural transformation from inclusion functor. (Note, an expanding functor is not the standard term in CT. We name it for the importance in consciousness studies, however, inclusion functor is a standard term in mathematics.) That is to say, an expansion functor, e, is an expanded form, or a version of the inclusion functor, i, transformed through some natural transformation.

### 3.2. Functor, Natural Transformation, and Consciousness

With the concepts of inclusion and expansion functors and natural transformation, we can now propose to provide a more explicit relationship between the neural activity N to the anatomical structure, where N is embedded, which is a necessary step to go beyond the traditional NCC approach. In this paper, we use the inclusion functor, i, as the basis and expansion functors as its expanded version, to stress the importance of the idea that expansion functors are the variations from the functor, i, as the basis through some natural transformation.

In the next section, we point out that some essential aspects of IIT and TTC can be captured by a considering different versions of expanding functors generated from the inclusion functor. We show that IIT and TTC distinguish between inclusion and expansion functor. As in the case of N0 and N1, interestingly, we will see that IIT and TTC can possibly incorporate inclusion functors, expansion functors, and natural transformations between them in different manners. Regardless of the specifics of the theories, we argue that these concepts of functor and natural transformation are some of the missing components of traditional NCC research. 

### 3.3. Functors and Natural Transformations in IIT and TTC

#### 3.3.1. Functors and Natural Transformations in IIT

Let us first reinterpret some aspects of IIT in CT, especially with the concepts of inclusion functor, expansion functors, and natural transformations between them. A critical concept in IIT, the amount of integrated information, *φ* or small phi, can be interpreted as the “difference” between the actual and the (minimally) disconnected network [59,60,75]. This can be captured by CT concepts of inclusion and expansion functors. The compositional aspects of IIT, or a set of small phis corresponds to a set of objects captured by these functors. The big phi, *Φ*, which corresponds to quantity (e.g., level) of consciousness, or system level integration, can now be interpreted as a natural transformation.

Let us unpack the above statements. As we explained in Figure 1 for IIT category, we consider category, N0, composed of objects (the actual network with TPM) and arrows, which decompose the system. We also consider another category, N1, composed of all possible networks with TPM and arrows. In addition to decomposition arrows, N1 is equipped with disconnection arrows. Obviously, N0 is included by N1. Inclusion functor, *i*, finds the objects and arrows in N1 that correspond to those in N0.

**Figure 7:**
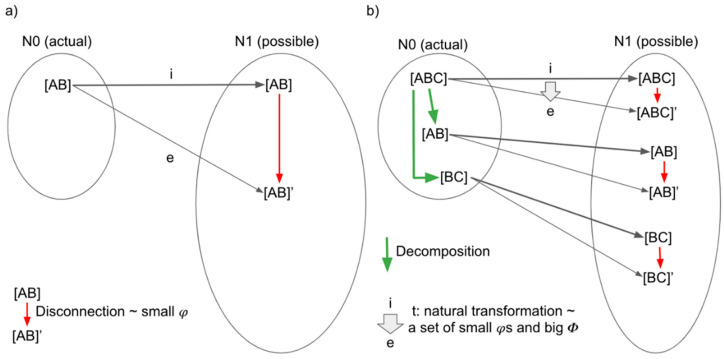
(**a**) Inclusion functor, i, expansion functor, e, in the IIT category N0 (actual) and N1 (all possible). Objects in N0 and N1 (e.g., [AB]) are a network with TPM, and arrows in N0 and N1 are manipulation of network/TPM that is allowed in IIT. Within N0, we consider only decomposition arrows. N1 is enriched by additional disconnection arrows that represent an operation that finds a “minimally disconnected” network with TPM within N1. An expansion functor, e, finds the minimally disconnected network (e.g., [AB]’) of the original network (e.g., [AB]), as well e also preserves the structure of N0, and qualifies as a functor. A red arrow within N1 that goes from the actual to the minimally disconnected network corresponds to integrated information, *φ*. (**b**) Considering decomposition arrows in N0 allows N0 to consist of a powerset of the network. If natural transformation, t, from the inclusion to the expansion functor exists, t gives us a power set of *φ*’s, the original and the minimally disconnected network with TPMs. This corresponds to system level integration, *Φ*.

Now, we define an expansion functor, *e*, as the one that finds the minimally disconnected version of the original network in N1 (Figure 7a).

Together with the inclusion functor, the expansion functor from the original network and TPM now gives us a set of small phis. Not only the original network (e.g., ABC) but also its subnetwork components (e.g., AB and BC) have corresponding small phi, which is derived by corresponding disconnection arrows in N1.

Now, we assume there is a natural transformation between inclusion and expansion functors. Then, a set of small phis is obtained by a natural transformation, *t*, between the inclusion and the expansion functors. This set can quantify integration at the system level, which corresponds to what IIT calls *Φ* or big phi. The concept of natural transformation clarifies the essence of IIT. IIT is a theory that proposes a set of small phis and a big phi, which corresponds to quality (e.g., qualia, contents) and quantity (e.g., level) of consciousness, respectively. (Here, what we propose is a mapping from a set of small phis (with their structural relation taken into account) into a scalar value of a big phi. This can include further operation of system-level disconnection, which we will not introduce here (See [53] for details). The nature of this mapping cannot be captured by a standard notion of multivariate function, which maps structure-less objects into a single object. What we need is a more flexible notion that takes the structure of small phis to relate it to a big phi (which involves system-level disconnection). All of these computational steps can be simply represented as an arrow in CT.)

**Figure 8:**
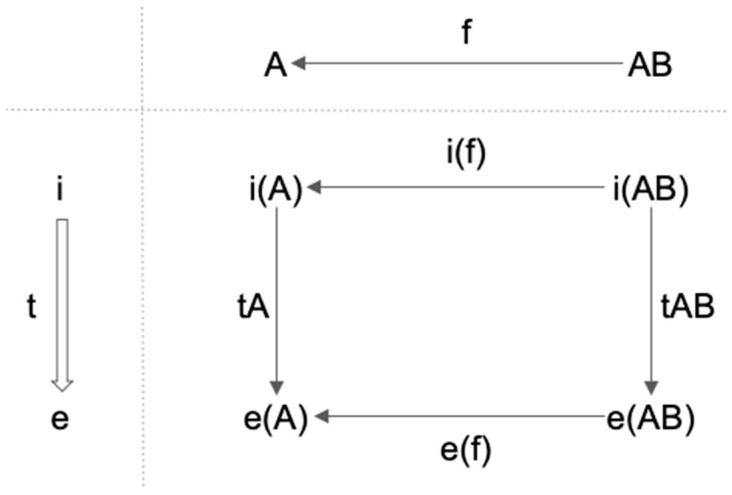
Natural transformation, t.

Does a natural transformation, t, really exist? We consider it in Figure 8. If t qualifies as a natural transformation, f, that is, a decomposition arrow from AB to A in N0 (or i(f) from i(AB) to i(A) in N1) has to correspond to a decomposition arrow in N1 from AB’ = e(AB) to A’ = e(A). As far as we know (including our personal communication with Dr. Masafumi Oizumi), IIT has never considered a precise mathematical formulation between the disconnected networks such as this. Thus, while we know that some kind of relationship exists between e(AB) and e(A), at this point, we believe that the operations that are used to decompose AB into A (i.e., f) cannot be directly applied to the disconnected AB’ into A’, at least under the IIT 3.0.

Here, let us briefly remark a potential consequence of the existence of a natural transformation. If one can describe the decomposition arrow between the disconnected networks in a formal mathematical relationship, which parallels the decomposition arrow between the original networks, then we can prove the existence of a natural transformation between inclusion and expansion functor. Mathematically, this guarantees the possibility of building up a larger network by considering a larger context (say, adding C into AB) in IIT. IIT papers, according to our understanding, have been so far mute on the possibility or limitation of this “reverse-reductionism” approach. Intuitively, however, the role of AB among ABC should be similar to the role of AB among ABCD (to some extent). Our preliminary results indeed suggest this may be the case, when integrated information is computed from the neural recording data [76]. If it is indeed the case that we can reverse-reductionistically understand the whole by building up and pasting many parts of the systems (potentially using presheaf theory [77]), then this approach may make IIT more mathematically tractable. 

Nevertheless, it is totally possible that there is no formal arrow like e(f). If that is the case, it practically means that the integrated information of a part of the system can completely and unpredictably change based on the way it is embedded in the context. This may reveal an extreme holistic property of the IIT. Given the phenomenological axiom of compositionality in IIT, however, we surmise that such a result probably requires a revision of the postulate of the IIT. This conjecture, a necessity and potential consequence of consideration between the disconnected networks, is a direct consequence of considering IIT from the CT perspective, which may prove useful in future mathematical examinations of IIT.

In summary, the category theoretic reinterpretation of IIT tells us that to construct quantitative theory of consciousness, consideration of the relation between actual and possible is necessary. More precisely, expanding functor, e (as a mapping towards a set of disconnected networks), in relation to inclusion functor needs to be considered. In terms of category theory, natural transformation from i to e provides us a set of small phis, or integrated information, which characterize quality of consciousness, and a big phi, the system level integration or quantity of consciousness. Quality and quantity of consciousness in IIT amounts to the quantitative evaluation on the natural transformation from i to e (if it exists). (We do not foresee that CT will directly prescribe or improve the detailed computational steps of IIT in this step as well. That will require different mathematical tools.) As we have defined, a natural transformation is a system of arrows indexed by objects which satisfies certain consistency condition, which requires further investigation.

#### 3.3.2. Functor and Natural Transformations in TTC

Now, TTC is compatible with the concepts of inclusion and expansion functors and a natural transformation, as TTC also emphasizes the need for conceiving the relationship between prestimulus activity (N0) and poststimulus activity (N1) in terms of integration (but not in the sense used in IIT). However, unlike IIT, TTC again emphasizes the dynamic, for example, temporospatial mechanisms that are supposedly underlying the relationship between pre- and poststimulus activity including their integration. 

To be more specific, an inclusion functor, i, from N0 to N1 would correspond to a mapping from the pre- to poststimulus neural activity without sensory input (or any other perturbation). An expansion functor, e, would correspond to a mapping from the pre- to poststimulus neural activity with a specific sensory input (or any other perturbation). Expansion functors, therefore, are a family of functors. Natural transformation between i and e describes relationships among all possible consequences of perturbations.

Traditional models presuppose that stimulus-induced activity as related to external stimuli is simply added to, and thus supervenes on the ongoing internal neuronal activity and this amounts to additive rest-stimulus interaction [78,79,80,81,82,83]. In contrast, recent findings suggest nonadditive interaction between pre- and poststimulus activity levels as based on EEG [63], fMRI [65,68,84,85], and computational modeling [86].

In the case of nonadditive interaction, the poststimulus activity is not simply added on or supervenes upon the prestimulus activity level. Instead, the level of prestimulus activity exerts a strong impact on the level of subsequent poststimulus activity. In terms of the response amplitude, low prestimulus activity levels lead to relatively higher poststimulus activity levels than high prestimulus activity levels [84,85,87]. Importantly, recent studies in MEG [72,73] and fMRI [87] demonstrate that prestimulus variance and its nonadditive impact on poststimulus amplitude and variance are related to conscious contents [70,72,73,88] and the level and state of consciousness [87]. Most interestingly, a recent study demonstrated that prepoststimulus variance changes are accompanied by the Lempel–Zev complexity (LZC) in the prestimulus interval [89,90]. LZC is used to compute the perturbational complexity index in a TMS-EEG experiment [91]. PCI is inspired by IIT as a proxy of integrated information and as a measure of level of consciousness. While how integrated information relates to PCI is unclear at this point, it raises a possible link between the nonadditive dynamics of prepoststimulus interaction, as pointed outed in TTC, with integrated information in IIT.

Another point on the importance of inclusion functor, exclusion functor, and natural transformation between them in the context of TTC is the importance of N1 (poststimulus activity) in relation to N0 (prestimulus activity) (N1 includes a larger set of activities as it refers to poststimulus activity which includes the prestimulus activity and, more specifically, how the prestimulus shapes or constrains the possible poststimulus activity. Moreover, N1 includes all potential poststimulus activity, which would include an actual prestimulus activity.). As the reviewed empirical evidence suggests, poststimulus activity (N1), reflecting the processing of the contents themselves, is not sufficient to explain any particular phenomenology, p, on its own. In addition to poststimulus activity (N1), prestimulus activity (N0) and its dynamics is necessary as N0 strongly affects and modulates how the subsequent N1 is processed. As such, TTC claims “consciousness does not come with the contents themselves” [6]. Instead, TTC suggests that consciousness is associated with the contents rather than coming with the contents themselves [3,21]. Empirically, this means that the focus shifts from the neural activity in the poststimulus period to the prestimulus activity and how it interacts with the stimulus, for example, the nonadditive dynamics of pre- and poststimulus interaction. Mathematically, that very same dynamic of nonadditive prepoststimulus interaction can be well formalized by the, here, assumed natural transformation from inclusion functor to expansion functor.

Our mathematical approach to especially TTC is compatible with the dynamic system accounts of the phenomenal features of consciousness. In a nutshell, TTC claims the need to extend the objects in order for them to be associated with consciousness [62] which is possible within the context of a dynamical activity space as characterized by a multitude of possible trajectories exhibiting temporospatial dynamics [30,34,35]. The TTC now claims that such dynamical extension is mediated by the interaction of the temporospatial features between neural states, for example, N0 and N1, and the respective object. Due to nonadditive prepoststimulus interaction, the object is thereby temporospatially extended in a virtual way, for example, temporospatial extension, by means of which the object can become consciously experienced [62]. Accordingly, the temporospatial extension of the temporospatial features of the object (e.g., N0 and N1) allows transforming neural states into phenomenal states, for example, natural transformation in the terms of CT. The temporospatial features (e.g., dynamical features), then, provide what has recently been described as “common currency” of neural and phenomenal features [62].

Moreover, in the context of TTC, natural transformation is a core issue. The TTC raises the question of how neuronal activity is transformed into phenomenology. For that, the TTC assumes that the interaction between prestimulus activity, as reflecting the brain’s ongoing dynamics, impacts and constrains its interaction with the external stimulus that may form the object of consciousness. Mechanistically, the TTC assumes that the way prestimulus activity constrains poststimulus activity is central for associating the external stimulus with consciousness. That leaves open though how neuronal activity of stimulus-induced activity transforms into phenomenology. This is the moment where TTC turns to CT and, more specifically, its concept of natural transformation. By formalizing the interaction between pre- and poststimulus activity in terms of the inclusion functor, CT links the neuronal mechanisms of prepoststimulus interaction with natural transformation. More generally, we assume that natural transformation is not just a matter specific to TTC but a more general and basic problem and a question that neuroscience needs to raise (i.e., How does neuronal activity transform into phenomenology?). As we elaborate in this paper, different answers can be given to that question, that is, either by integration of information (IIT) temporospatial dynamic (TTC).

## 4. Conclusions

In this paper, we introduced category theory (CT) to account and formalize the relationship between the neuronal (N0 and N1) and phenomenal (P) domains in the neuroscience of consciousness. Specifically, we introduced four fundamental concepts in CT (category, inclusion, expansion functors, and, most importantly, natural transformation) in the context of two major neuroscientific theories of consciousness, for example, IIT and TTC. Now, we briefly review some major implications for our search of the NCC in general in the future neuroscientific studies of consciousness.

The first point we made was that we need to distinguish between two different neuronal categories, N0 and N1, which IIT and TTC implicitly have proposed. This approach seems to solve a difficulty in traditional NCC research, which implicitly assumes the anatomical frame when it considers one specific neuronal state and its corresponding one specific phenomenal state. By explicitly considering two neural categories, both IIT and TTS consider N0 (a particular neural state) as embedded with N1 (all possible states), which is constrained by the anatomical reference frame.

The second point, which is even more important, was a shift of focus from the relationship between neuronal and phenomenal states, as promoted by the traditional NCC approach, to the relationship and, specifically, a particular form of relationship or interaction between two neuronal category (N0 and N1) as central for yielding consciousness. This emphasis of the relationship can be framed as natural transformation between inclusion and expansion functors. Addressing the same question, IIT and TTC provide different answers, for example, a set of small phis or integrated information in IIT and a nonadditive interaction in TTC. Through the lens of natural transformation, our reanalysis of IIT suggests a pathway to a novel reverse reductionistic approach in the empirical computation of an integrated information structure for a whole large system based on its subsystem. IIT (as formulated by [53]), PLoS Comp in particular, does not allow any inference of how a subset of neurons and mechanisms would contribute to the whole without IIT analyses on the whole (including appropriate search for so-called “complex”, decomposition, and disconnection at all levels), which makes the analysis intractable, empirically. Our reverse reductionism idea, however, is to allow such inference, by starting the analysis of the local subset of the neurons without any context, and to extend it to the case where it is embedded in the larger network. If a natural transformation exists, the small phi structures should be retained in some form. While the current IIT3.0 prohibits the existence of natural transformation and reverse reductionism, this does not mean that our approach is wrong, and possibly IIT can be modified to allow natural transformation to exist. Yet another possibility is that while a natural transformation does not exist at a strict sense, some types of approximation (e.g., atomic partition rather than MIP, mutual information rather than integrated information) may allow a natural transformation to exist. With such an approximation, the reverse reductionistic approximation may turn out to be powerful (see a similar idea on submodularity to approximate MIP in [54]).

Taken together, we conclude that CT provides the mathematical tools to formalize the relationship between the neuronal and the phenomenal domains and to give a blueprint on how to extend it beyond the traditional NCC approaches. In the context of IIT, a mathematical investigation on the existence of natural transformation between inclusion and expansion functors can be a potentially fruitful investigation, as it may allow a reverse reductionistic approach to understand a large network to overcome the fundamental difficulty in IIT. In the context of TTC, CT can extend the concept of nonadditivity into temporospatial dynamics.

As such, we conclude that the introduction of CT in the study of neural correlates of consciousness awaits further fruitful theoretical development, with its potential to connect or translate across different theories of consciousness, which we could not mention in this paper (e.g., the global neuronal workspace theory (GNWT) [16,17,18,19], higher order theories of consciousness [22,23], recurrent processing theory [24], operational space and time [25], neural synchrony [26], and social and attention schema theory [27]). Comparison of the theories through CT, as we did for IIT and TTC here, may inspire development of an entirely novel approach to connect neuronal and phenomenal domains in a formal and mathematical way. (We also note that our program is a practical and yet mathematically well-founded formalism to disprove IIT (if IIT is wrong), unlike other types of criticisms of IIT on philosophical or other unclear grounds. Rather than starting from “identity” between MICS and P, as the original IIT assumes, we propose to leave the relationship between MICS and P as something to be tested and established. Our program will provide two concrete strategy. First, to test if there exists a functor from phenomenal (P) to neural (N0, N1) or MICS, and second, to test if there exists a functor from neural (N0, N1) or MICS to phenomenal (P). Note that this program does not have to start from the entire conscious experience, which makes the research program intractable. Unlike the IIT program, our program can be applied to a subset of phenomenal domain.)

Importantly, the implications of our approach extend beyond the merely theoretical understanding of neurophenomenal relationship [74] to practical and clinical application. First, based on mathematical formalization in the terms of CT with a focus on possible (rather than actual) states operating as inclusion and exclusion functors, our approach opens the door for engineers to reverse-engineer conscious artifacts. For example, if a reverse reductionistic approach can work in IIT, it can prescribe a recipe for how to generate a potentially large integrated information system by combining locally highly integrated information systems (but also see Aaronson’s blog and Tononi’s reply on this type of argument https://www.scottaaronson.com/blog/?p=1823). Secondly, assuming TTC is the right way to understand consciousness, then knowledge of how expansion functor is supported by neuronal mechanisms to realize nonadditive rest–stimulus interaction may point to novel therapeutic techniques and anatomical targets for brain stimulation. Such stimulation techniques may be able to restore the brain functions underlying loss of consciousness in coma patients and altered consciousness in psychiatric patients suffering from schizophrenia [92,93], bipolar disorder (with mania and depression) [6,94], and major depressive disorder [5]. These patients show changes in their spontaneous activity which, according to TTC, may be related to the integration between inclusion and expansion functor. An exact mathematical description may allow development of computational models of that interaction which could serve as basis for developing individualized mechanism-based stimulation therapy such as with either deep brain stimulation or transcranial magnetic stimulation.

## References

[1] Levine J. (1983). Materialism and qualia: The explanatory gap. Pac. Philos. Q..

[2] Chalmers D.J. (2000). What Is a Neural Correlate of Consciousness? Neural Correlates of Consciousness: Empirical and Conceptual Questions.

[3] Northoff G. (2014). Unlocking the Brain: Volume II: Consciousness.

[4] Churchland P. (2002). Brain-Wise.

[5] Northoff G. (2016). Neuro-Philosophy and the Healthy Mind: Learning from the Unwell Brain.

[6] Northoff G. (2018). The Spontaneous Brain. From Mind-Body Problem to World-Brain Problem.

[7] Searle J.R. (2004). Mind: A Brief Introduction.

[8] Arzi-Gonczarowski Z. (1999). Perceive this as that—Analogies, artificial perception, and category theory. Ann. Math. Artif. Intell..

[9] Crick F., Koch C. (2003). A framework for consciousness. Nat. Neurosci..

[10] De Graaf T.A., Hsieh P.J., Sack A.T. (2012). The ‘correlates’ in neural correlates of consciousness. Neurosci. Biobehav. Rev..

[11] Koch C. (2004). The Quest for Consciousness.

[12] Northoff G. (2014). Unlocking the Brain: Volume I: Coding.

[13] Koch C., Massimini M., Boly M., Tononi G. (2016). Neural correlates of consciousness: Progress and problems. Rev. Neurosci..

[14] Tononi G. (2004). An information integration theory of consciousness. BMC Neurosci..

[15] Tononi G., Boly M., Massimini M., Koch C. (2016). Integrated information theory: From consciousness to its physical substrate. Nat. Rev. Neurosci..

[16] Dehaene S., Charles L., King J.R., Marti S. (2014). Toward a computational theory of conscious processing. Curr. Opin. Neurobiol..

[17] Dehaene S., Changeux J.P. (2011). Experimental and theoretical approaches to conscious processing. Neuron.

[18] Dehaene S., Naccache L. (2001). Towards a cognitive neuroscience of consciousness: Basic evidence and a workspace framework. Cognition.

[19] Baars B.J. (2005). Global workspace theory of consciousness: Toward a cognitive neuroscience of human experience. Prog. Brain Res..

[20] Northoff G. (2013). What the brain’s intrinsic activity can tell us about consciousness? A tri-dimensional view. Neurosci. Biobehav. Rev..

[21] Northoff G., Huang Z. (2017). How do the brain’s time and space mediate consciousness and its different dimensions? Temporospatial theory of consciousness (TTC). Neurosci. Biobehav. Rev..

[22] Lau H., Rosenthal D. (2011). Empirical support for higher-order theories of conscious awareness. Trends Cogn. Sci..

[23] Rosenthal D.M. (2000). Metacognition and higher-order thoughts. Conscious. Cogn..

[24] Lamme V.A., Roelfsema P.R. (2000). The distinct modes of vision offered by feedforward and recurrent processing. Trends Neurosci..

[25] Fingelkurts A.A., Fingelkurts A.A., Neves C.F. (2010). Natural world physical, brain operational, and mind phenomenal space-time. Phys. Life Rev..

[26] Engel A.K., Singer W. (2001). Temporal binding and the neural correlates of sensory awareness. Trends Cogn. Sci..

[27] Graziano M.S., Kastner S. (2011). Human consciousness and its relationship to social neuroscience: A novel hypothesis. Cogn. Neurosci..

[28] Tsuchiya N., Taguchi S., Saigo H. (2016). Using category theory to assess the relationship between consciousness and integrated information theory. Neurosci. Res..

[29] Stanley R.P. (1999). Qualia space. J. Conscious. Stud..

[30] Yoshimi J. (2011). Phenomenology and connectionism. Front. Psychol..

[31] Hoffman W.C. (1980). Subjective geometry and geometric psychology. Math. Model..

[32] Hoffman W.C. (1966). The Lie algebra of visual perception. J. Math. Psychol..

[33] Palmer S.E. (1999). Color, consciousness, and the isomorphism constraint. Behav. Brain Sci..

[34] Prentner R. (2019). Consciousness and topologically structured phenomenal spaces. Conscious. Cogn..

[35] Fekete T., Edelman S. (2011). Towards a computational theory of experience. Conscious. Cogn..

[36] Eilenberg S., MacLane S. (1945). Relations between homology and homotopy groups of spaces. Ann. Math..

[37] Baez J.C., Stay M. Physics, Topology, Logic. and Computation: A Rosetta Stone. https://arxiv.org/abs/0903.0340.

[38] Ehresmann A.C., Vanbremeersch J.P. (1987). Hierarchical evolutive systems: A mathematical model for complex systems. Bull. Math. Biol..

[39] Ehresmann A.C., Vanbremeersch J.P. (1997). Information processing and symmetry-breaking in memory evolutive systems. Biosystems.

[40] Ehresmann A.C., Gomez-Ramirez J. (2015). Conciliating neuroscience and phenomenology via category theory. Prog. Biophys. Mol. Biol..

[41] Healy M.J., Caudell T.P., Goldsmith T.E. (2008). A Model of Human Categorization and Similarity Based Upon Category Theory.

[42] Phillips S., Wilson W.H. (2010). Categorial compositionality: A category theory explanation for the systematicity of human cognition. PLoS Comput. Biol..

[43] Phillips S., Wilson W.H. (2016). Systematicity and a categorical theory of cognitive architecture: Universal construction in context. Front. Psychol..

[44] Allison T., Ginter H., McCarthy G., Nobre A.C., Puce A., Luby M., Spencer D.D. (1994). Face recognition in human extrastriate cortex. J. Neurophysiol..

[45] Baroni F., van Kempen J., Kawasaki H., Kovach C.K., Oya H., Howard M.A., Adolphs R., Tsuchiya N. (2017). Intracranial markers of conscious face perception in humans. Neuroimage.

[46] Kanwisher N., Yovel G. (2006). The fusiform face area: A cortical region specialized for the perception of faces. Philos. Trans. R. Soc. Lond. B Biol. Sci..

[47] Tong F., Nakayama K., Vaughan J.T., Kanwisher N. (1998). Binocular rivalry and visual awareness in human extrastriate cortex. Neuron.

[48] Rangarajan V., Hermes D., Foster B.L., Weiner K.S., Jacques C., Grill-Spector K., Parvizi J. (2014). Electrical stimulation of the left and right human fusiform gyrus causes different effects in conscious face perception. J. Neurosci..

[49] Chialvo D.R. (2010). Emergent complex neural dynamics. Nat. Phys..

[50] Rees G., Friston K., Koch C. (2000). A direct quantitative relationship between the functional properties of human and macaque V5. Nat. Neurosci..

[51] Balduzzi D., Tononi G. (2009). Qualia: The geometry of integrated information. PLoS Comput. Biol..

[52] Tononi G. (2010). Information integration: Its relevance to brain function and consciousness. Arch. Ital. Biol..

[53] Oizumi M., Albantakis L., Tononi G. (2014). From the phenomenology to the mechanisms of consciousness: Integrated Information Theory 3.0. PLoS Comput. Biol..

[54] Hidaka S., Oizumi M. (2018). Fast and exact search for the partition with minimal information loss. PLoS ONE.

[55] Toker D., Sommer F.T. (2019). Information integration in large brain networks. PLoS Comput. Biol..

[56] Tsuchiya N., Andrillon T., Haun A. (2019). A reply to “the unfolding argument”: Beyond functionalism/behaviorism and towards a truer science of causal structural theories of consciousness. PsyArXiv.

[57] Awodey S. (2010). Category Theory.

[58] Haun A.M., Oizumi M., Kovach C.K., Kawaski H., Oya H., Howard M.A., Adolphs R., Tsuchiya N. (2017). Conscious perception as integrated information patterns in human electrocorticography. eNeuro.

[59] Oizumi M., Tsuchiya N., Amari S.I. (2016). Unified framework for information integration based on information geometry. Proc. Natl. Acad. Sci. USA.

[60] Tegmark M. (2016). Improved measures of integrated information. PLoS Comput. Biol..

[61] Northoff G. (2017). Paradox of slow frequencies—Are slow frequencies in upper cortical layers a neural predisposition of the level/state of consciousness (NPC)?. Conscious. Cogn..

[62] Northoff G. (2019). The anxious brain and its heart—Temporal brain-heart de-synchronization in anxiety disorders. J. Affect. Disord..

[63] He B.J., Zempel J.M. (2013). Average is optimal: An inverted-U relationship between trial-to-trial brain activity and behavioral performance. PLoS Comput. Biol..

[64] Northoff G., Qin P., Nakao T. (2010). Rest-stimulus interaction in the brain: A review. Trends Neurosci..

[65] Huang Z., Zhang J., Longtin A., Dumont G., Duncan N.W., Pokorny J., Qin P., Dai R., Ferri F., Weng X. (2017). Is There a Nonadditive Interaction Between Spontaneous and Evoked Activity? Phase-Dependence and Its Relation to the Temporal Structure of Scale-Free Brain Activity. Cereb. Cortex..

[66] Boly M., Phillips C., Tshibanda L., Vanhaudenhuyse A., Schabus M., Dang-Vu T., Moonen G., Hustinx R., Maquet P., Laureys S. (2008). Intrinsic brain activity in altered states of consciousness: How conscious is the default mode of brain function?. Ann. N. Y. Acad. Sci..

[67] Hesselmann G., Kell C.A., Eger E., Kleinschmidt A. (2008). Spontaneous local variations in ongoing neural activity bias perceptual decisions. Proc. Natl. Acad. Sci. USA.

[68] Sadaghiani S., Hesselmann G., Friston K.J., Kleinschmidt A. (2010). The relation of ongoing brain activity, evoked neural responses, and cognition. Front. Syst. Neurosci..

[69] Sadaghiani S., Hesselmann G., Kleinschmidt A. (2009). Distributed and antagonistic contributions of ongoing activity fluctuations to auditory stimulus detection. J. Neurosci..

[70] Arazi A., Censor N., Dinstein I. (2017). Neural Variability Quenching Predicts Individual Perceptual Abilities. J. Neurosci..

[71] Bai Y., Nakao T., Xu J., Qin P., Chaves P., Heinzel A., Duncan N., Lane T., Yen N.S., Tsai S.Y. (2011). Resting state glutamate predicts elevated prestimulus alpha during self-relatedness: A combined EEG-MRS study on “rest-self overlap”. Soc. Neurosci..

[72] Baria A.T., Maniscalco B., He B.J. (2017). Initial-state-dependent, robust, transient neural dynamics encode conscious visual perception. PLoS Comput. Biol..

[73] Liu C.H., Ma X., Song L.P., Fan J., Wang W.D., Lv X.Y., Zhang Y., Li F., Wang L., Wang C.-Y. (2015). Abnormal spontaneous neural activity in the anterior insular and anterior cingulate cortices in anxious depression. Behav. Brain Res..

[74] Northoff G., Wainio-Theberge S., Evers K. (2019). Is temporospatial dynamics the “common currency” of brain and mind? In Quest of “Spatiotemporal Neuroscience”. Phys. Life Rev..

[75] Oizumi M., Amari S., Yanagawa T., Fujii N., Tsuchiya N. (2016). Measuring integrated information from the decoding perspective. PLoS Comput. Biol..

[76] Leung A., Cohen D., van Swinderen B., Tsuchiya N. (2018). General anaesthesia reduces integrated information in flies. Monash Univ..

[77] Fong B., Spivak D.I. (2018). Seven Sketches in Compositionality: An Invitation to Applied Category Theory. https://arxiv.org/abs/1803.05316.

[78] Arieli A., Sterkin A., Grinvald A., Aertsen A. (1996). Dynamics of ongoing activity: Explanation of the large variability in evoked cortical responses. Science.

[79] Azouz R., Gray C.M. (1999). Cellular mechanisms contributing to response variability of cortical neurons in vivo. J. Neurosci..

[80] Fox M.D., Snyder A.Z., Zacks J.M., Raichle M.E. (2006). Coherent spontaneous activity accounts for trial-to-trial variability in human evoked brain responses. Nat. Neurosci..

[81] Fox M.D., Snyder A.Z., Vincent J.L., Raichle M.E. (2007). Intrinsic fluctuations within cortical systems account for intertrial variability in human behavior. Neuron.

[82] Fox M.D., Raichle M.E. (2007). Spontaneous fluctuations in brain activity observed with functional magnetic resonance imaging. Nat. Rev. Neurosci..

[83] Sylvester C.M., Shulman G.L., Jack A.I., Corbetta M. (2009). Anticipatory and stimulus-evoked blood oxygenation level-dependent modulations related to spatial attention reflect a common additive signal. J. Neurosci..

[84] Ferri F., Costantini M., Huang Z., Perrucci M.G., Ferretti A., Romani G.L., Northoff G. (2015). Intertrial variability in the premotor cortex accounts for individual differences in peripersonal space. J. Neurosci..

[85] Ferri F., Nikolova Y.S., Perrucci M.G., Costantini M., Ferretti A., Gatta V., Huang Z., Edden R.A.E., Yue Q., D’Aurora M. (2017). A Neural “Tuning Curve” for Multisensory Experience and Cognitive-Perceptual Schizotypy. Schizophr. Bull..

[86] Ponce-Alvarez A., He B.J., Hagmann P., Deco G. (2015). Task-driven activity reduces the cortical activity space of the brain: Experiment and whole-brain modeling. PLoS Comput. Biol..

[87] Huang Z., Zhang J., Wu J., Liu X., Xu J., Zhang J., Qin P., Dai R., Yang Z., Mao Y. (2018). Disrupted neural variability during propofol-induced sedation and unconsciousness. Hum. Brain Map..

[88] Schurger A., Sarigiannidis I., Naccache L., Sitt J.D., Dehaene S. (2015). Cortical activity is more stable when sensory stimuli are consciously perceived. Proc. Natl. Acad. Sci. USA.

[89] Wolff A., Di Giovanni D.A., Gómez-Pilar J., Nakao T., Huang Z., Longtin A., Northoff G. (2019). The temporal signature of self: Temporal measures of resting-state EEG predict self-consciousness. Hum. Brain Map..

[90] Wolff A., Gómez-Pilar J., Nakao T., Northoff G. (2019). Interindividual neural difference in moral decision-making are mediated by alpha power and delta/theta phase coherence. Sci. Rep..

[91] Bayne T. (2010). The Unity of Consciousness.

[92] Ebisch S.J.H., Gallese V., Salone A., Martinotti G., di Iorio G., Mantini D., Perrucci M.G., Romani G.L., Di Giannantonio M., Northoff G. (2018). Disrupted relationship between “resting state” connectivity and task-evoked activity during social perception in schizophrenia. Schizophr. Res..

[93] Northoff G., Duncan N.W., Hayes D.J. (2010). The brain and its resting state activity-experimental and methodological implications. Prog. Neurobiol..

[94] Martino D.J., Samame C., Strejilevich S.A. (2016). Stability of facial emotion recognition performance in bipolar disorder. Psych. Res..

